# PH-Responsive, Cell-Penetrating, Core/Shell Magnetite/Silver Nanoparticles for the Delivery of Plasmids: Preparation, Characterization, and Preliminary In Vitro Evaluation

**DOI:** 10.3390/pharmaceutics12060561

**Published:** 2020-06-17

**Authors:** Carlos M. Ramírez-Acosta, Javier Cifuentes, Maria Claudia Castellanos, Rodolfo José Moreno, Carolina Muñoz-Camargo, Juan C. Cruz, Luis H. Reyes

**Affiliations:** 1 Grupo de Diseño de Productos y Procesos (GDPP), Department of Chemical and Food Engineering, Universidad de los Andes, Bogotá 111711, Colombia; cm.ramirez10@uniandes.edu.co (C.M.R.-A.); rj.moreno@uniandes.edu.co (R.J.M.); 2 Department of Biomedical Engineering, Universidad de los Andes, Bogotá 111711, Colombia; jf.cifuentes10@uniandes.edu.co (J.C.); mc.castellanos10@uniandes.edu.co (M.C.C.); c.munoz2016@uniandes.edu.co (C.M.-C.)

**Keywords:** gene delivery, core-shell nanoparticles, pH-responsive polymer, magnetite

## Abstract

Over the past decade, gene therapies have attracted much attention for the development of treatments for various conditions, including cancer, neurodegenerative diseases, protein deficiencies, and autoimmune disorders. Despite the benefits of this approach, several challenges are yet to be solved to reach clinical implementation. Some of these challenges include low transfection rates, limited stability under physiological conditions, and low specificity towards the target cells. An avenue to overcome such issues is to deliver the therapies with the aid of potent cell-penetrating vectors. Non-viral vectors, such as nanostructured materials, have been successfully tested in drug and gene delivery. Here, we propose the development and in vitro evaluation of a nanostructured cell-penetrating vehicle based on core/shell, magnetite/silver nanoparticles. A subsequent conjugation of a pH-responsive polymer was used to assure that the vehicle can carry and release circular DNA. Additionally, the translocating peptide Buforin II was conjugated with the aid of a polyether amine polymer to facilitate translocation and endosome escape. The obtained nanobioconjugates (magnetite/silver-pDMAEMA-PEA-BUFII) were characterized by UV-Vis spectrophotometry, dynamic light scattering (DLS), thermogravimetric analysis (TGA), Fourier transform infrared spectroscopy (FTIR), scanning electron microscope equipped with energy dispersive spectroscopy (SEM+EDS), and transmission electron microscopy (TEM). They were also encapsulated in lecithin liposomes to form magnetoliposomes. The cell viability of Vero cells in the presence of the nanobioconjugates was above 95% and declined to 80% for the magnetoliposomes. The hemolytic tendency of nanobioconjugates and magnetoliposomes was below 10%, while the platelet aggregation approached that of the negative control (i.e., 35%). Cytoplasm coverage values of about 50% for both Vero and neuroblastoma cells confirmed significant cell penetration. Pearson’s correlation coefficients for both cell lines allowed us to estimate 20–40% colocalization of the nanobioconjugates with lysotracker green, which implied high levels of endosomal escape. The developed vehicles were also capable of loading around 16% of the added DNA and releasing such cargo with 8% efficiency. The developed nanoplatform holds a significant promise to enable highly efficient gene therapies as it overcomes some of the major issues associated with their eventual translation to the pre-clinical and clinical scale.

## 1. Introduction

The delivery of pharmacological compounds to achieve higher bioavailability represents one of the major challenges of modern pharmacology [1]. This is mainly due to the high selectivity and permeability of biological membranes that prevent the free bypass of such compounds [2]. As a result, only a small fraction of the delivered molecules reaches the site of action [1,2]. Some of the approaches to overcome this issue include viral vectors, micelles, solubility enhancement agents, encapsulation systems, and nanomaterials with different surface chemistries [3,4,5]. The viral vectors have demonstrated relatively high delivery efficiencies but received considerable criticism by the biomedical community due to safety issues [5,6]. The past two decades have served for the consolidation of nanomaterials as major workhorses for the development of much more potent vehicles for the transport and delivery of different types of pharmacological cargoes ranging from small synthetic molecules to nucleotide sequences [4,7]. In this regard, some of the delivered small molecules included oxorubicin, 5-fluorouracil, and daunorubicin for the treatment of cancer [8,9,10]. The last 5 years have also seen an exponential increase in the use of gene therapies based on gene edition such as CRISPR/Cas9, zinc finger nucleases (ZFNs), and transcription activator-like effector nucleases (TALENs). In particular, CRISPR/Cas9 appears superior due to specificity, ease of use, and cost-effectiveness [11,12]. Despite these benefits, the implementation of CRISPR/Cas9 at the clinical level is still challenging due to issues regarding off-target effects, mosaicism, and specificity [11,13,14]. In order to address the off-target effects and specificity, the first approach is the use of a delivery system capable of providing high penetration rates and homogeneous intracellular distributions only in a particular cell line while protecting the DNA from degradation.

Efficient delivery of both circular and linear nucleotide sequences has been achieved through two main strategies, namely, viral vectors and non-viral vectors [15]. Viral vectors have proven to be efficient due to their high transfection rates, persistent expression, and low cytotoxicity. These attributes make them suitable for gene therapy [11,16]. Among viral vectors, the most common are retroviruses, lentiviruses, and adeno-associated viruses. Retroviruses can integrate efficiently into the chromatin of target cells, which is ideal for increased efficiency [5]. Lentiviruses’ strategy is to increase the transfection rate as they rely on the machinery of the cell and can target non-dividing cells [11,17]. Finally, adeno-associated viruses have been mainly utilized for genome transduction, which targets both dividing and non-dividing cells [11,18]. Despite the advantages of these systems, viral vectors still have some safety issues to overcome, such as immunogenicity and oncogenicity [4,6].

Alternatively, the use of non-viral vectors in gene and drug delivery has significantly increased, thereby leading to several new approaches to targeted gene therapies in both dividing and non-dividing cells. The main three non-viral vectors are nanostructured materials, liposomes, and polymers [4,19]. Thus far, the most studied and successful materials for delivery vehicles are polymers due to their abilities to condense linear and circular DNA into nano-sized polyplexes through electrostatic interactions [20]. Additionally, extracellular fluids can quickly degrade them along with any residual DNA to make them easily excretable [20].

The remarkable richness of polymeric molecules has led to the development of a vast arsenal of homopolymers and copolymers capable of targeting specific cell lines and even responding to unique tissue and cellular environments of diseased organs [2]. Also, polymers are advantageous because they are highly tailorable and have the ability to self-assemble into different shapes [6]. For instance, recent developments have targeted HepG2, and A549 cell lines with polyamidoamine, PC-3 prostate cancer cell line with polyethyleneimine (PEI) plus a polyethyleneglycol (PEG) linker, studies which demonstrate the promising ability of the polymers for delivery purposes [21,22]. Additionally, You et al. [23] developed a pH-responsive polymeric system with the ability to not only penetrate cells but deliver a carDNA cargo once it is internalized. Despite the attractiveness of polymeric vehicles, they exhibit several limitations, including high cytotoxicity, low loading efficiency, and degradation in the extracellular environment [6,7]. Some strategies have been developed to address these issues, such as conjugation of peptides or aptamers to reduce cytotoxicity and increase specificity [6,22]. An additional group of non-viral vectors with appealing attributes is the micellar systems and liposomes. These vehicles primarily function as encapsulating systems where genes or proteins are protected from degradation in extracellular environments and can extend the circulation times [7,24]. Despite these potentially beneficial features, the liposomes and micelles exhibit some limitations such as transient gene expression, low transfection efficiency, and an increase of cytotoxicity with concentration due to the presence of cationic lipids [6,15].

Finally, the last family of vehicles with the potential for clinically relevant applications in the near future are those based on nanomaterials. Thus far, different families of nanomaterials have been tested for gene delivery, such as metallic oxides, carbon-based, and polymeric [20,25,26]. An attractive feature of nanomaterials is that not only the surface chemistry can be tuned, but also their 3D topologies and size distribution [27,28,29]. In this regard, by controlling the synthesis conditions, it is possible to alter the assembly processes of precursors to obtain the desired physicochemical properties [30]. Moreover, it is possible to adjust the surface properties of the materials to respond to environmental changes by conjugating different chemistries without significantly altering their biocompatibility [20,26,31]. As a result, it is possible to engineer multifunctional nanovehicles that can be tuned to interact with tissues and cell lines in a specific manner, bypassing biological barriers (e.g., cell membranes, endosomes, and connective tissue) [3,7,32]. Recent examples of successful nucleotide cargoes include, among many others, gold nanoparticles for the delivery of Cas9 and a DNA-HS to Human H9 embryonic stem cells, and silica nanoparticles for the delivery of β-galactosidase expression plasmid pCMVb to Cos-1 cells [32,33]. Another interesting example was presented by Zhuang Liu et al., who delivered siRNA molecules in vitro with the aid of carbon nanotubes to silence the CD4 gene associated with HIV-specific cell-surface receptors [34]. Additionally, recent reports described the use of gold nanoparticles for the delivery of clustered regularly interspaced short palindromic repeats CRISPR/Cas9 for the editing of the CXCR4 gene or the dystrophin gene, associated with Duchenne muscular dystrophy (DMD) disease [33].

Despite the tremendous opportunities of non-viral vectors, some effort is still needed to understand their transport and fate within the human body [13,20]. The fate of the non-viral vectors in the human body is highly dependent on the charge and size of the vehicle, as well as on the specific targeted cells [2,7]. For instance, positively charged vectors tend to move biodistribution closer to the heart and lungs, while the ones negatively charged are preferentially distributed in proximity to the liver. Moreover, negative charges tend to prolong the blood residence and consequently, the systemic circulation time [2]. The chemistry also affects the clearance and elimination of non-viral vectors such that the vehicles with lower aqueous solubility tend to have faster clearance [7]. Size is also a decisive factor as particles with larger sizes tend to be clear by hepatic filtration or bioaccumulate in the liver instead of being eliminated [2,7]. Even though the clearance mechanism and engineering of non-viral vectors have been widely studied to address particular delivery needs, novel chemistries and multifunctional systems emerge on a daily basis, which limits the ability to make sound predictions of the exact fate of a vehicle in the body [3]. Nevertheless, non-viral vectors have achieved an increase in the delivery efficiencies throughout time, which makes them promising candidates to enable high-bioavailability therapies.

Accordingly, this work, therefore, aims at designing, manufacturing, characterizing, and testing a nanovehicle capable of loading and delivering plasmid DNA with the ultimate goal of enabling gene therapies. To achieve this, here we proposed to synthesize a nanoscale core/shell system of magnetite/silver functionalized with (poly (2-dimethylamino) ethyl methacrylate) methyl chloride (pDMAEMA), a polymer with the ability to form complexes with DNA [23,26]. Moreover, the selected polymer exhibits pH-responsiveness, which makes it attractive for intracellular delivery, under the reducing conditions of the cytoplasm, and even endosomal escape [26,30,32]. Based on our previous work, we also conjugated the antimicrobial peptide Buforin II to increase translocation and endosomal escape efficiencies even further [35]. Finally, the developed nanovehicles were encapsulated into liposomes to increase stability and cell-penetration efficiency as well as potentially improve systemic circulation time and reduce immune responses in the eventual case of reaching in vivo applications [4,15]. We characterized the obtained nanovehicles with the aid of thermal, spectroscopy, and microscopy techniques. Also, we conducted DNA loading and delivery experiments, and finally, we evaluated their biocompatibility and ability to escape endosomes in two different cell lines. Our findings suggest that the developed nanovehicles are capable of loading significant amounts of plasmid DNA, penetrate mammalian cells without detrimentally impacting viability, and escape endosomes quite efficiently. Further experiments will be focused on delivering CRISPR/Cas9 therapies in vitro and in vivo to test transfection efficiencies and the gene-editing effectiveness.

## 2. Materials and Methods

### 2.1. Magnetite Core and Silver Shell Synthesis

The magnetite nanoparticles were synthesized by co-precipitating 500 mM Iron (II) chloride (J. T. Baker, USA) and 250 mM Iron (III) chloride (Merck, Kenilworth, NJ, USA), in a 2:1 molar ratio, in the presence of a 5 M NaOH (PanReac AppliChem, Darmstadt, Germany) solution at 90 °C. The iron chloride solutions were dissolved in Milli-Q water and dripped over the sodium hydroxide solution at a rate of 5 mL/min. Once the reaction took place, the nanoparticles were resuspended using a sonic tip at a frequency of 20 kHz and precipitated with a neodymium magnet to remove the supernatant. The nanoparticles were washed by repeated cycles of Milli-Q water aided by magnetic separation.

**Figure 1:**
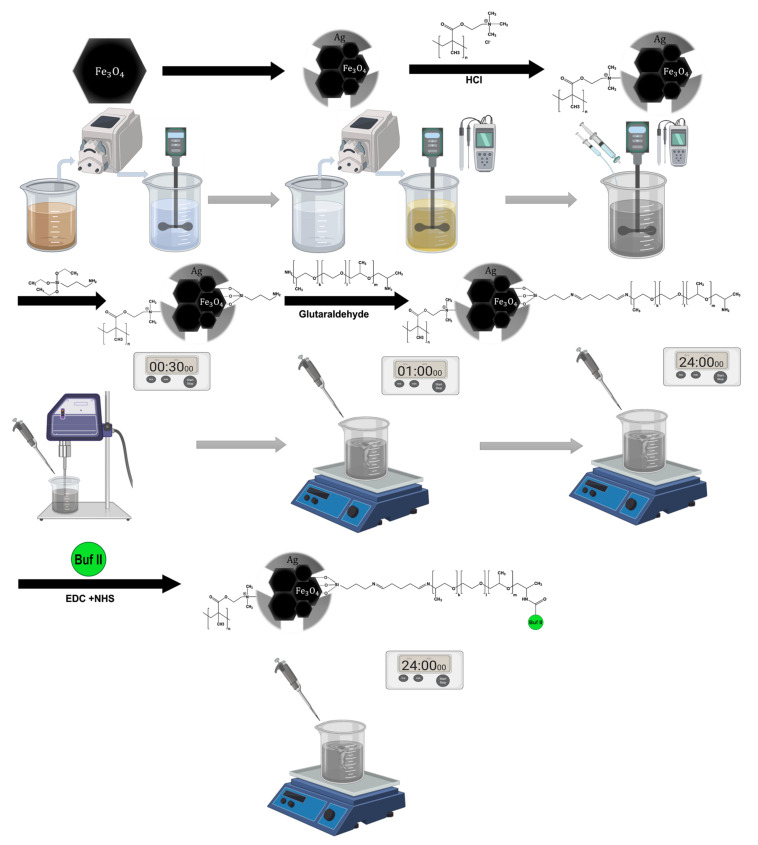
Graphical methodology. The figure represents the steps followed in the synthesis and immobilization of the vehicle. First, magnetite was synthesized using the co-precipitation method. Second, the silver coating was made by using a redox reaction with control of the pH. Third, the pDMAEMA was immobilized on the core/shell magnetite/silver surface, stirring, and the pH was carefully controlled. Finally, the conjugation of Buforin II, using the Polyether Amine, Jeffamine^®^, as a surface spacer and APTES to anchor the molecules on the surface.

The silver shell was formed by a redox reaction of 1 mM silver nitrate (Merck, Kenilworth, NJ, USA) in 20% (*w*/*v*) in an aqueous solution of honey (from farmer’s market, under the Colombian regulatory framework and used without prior purification) as a reducing agent. Six mL of magnetite solution was added to the honey solution at a rate of 5 mg/mL under constant stirring while maintaining pH above 4 with the aid of a 1 M NaOH until a complete reaction of the silver nitrate precursor was achieved. Subsequently, the pH was adjusted to 8 using NaOH (1M). The experimental set up is shown in Figure 1. The magnetite-silver nanoparticles were washed using the same procedure described above, but, in this case, the MilliQ water was heated up to 75 °C to remove excess reagents. Magnetic precipitation excludes free silver nanoparticles from those with a magnetic core.

### 2.2. Conjugation of the PH-Responsive Polymer DMAEMA on Magnetite/Silver Nanoparticles

Prior to conjugating the pH-responsive polymer, poly (2-dimethylamino) ethyl methacrylate) methyl chloride quaternary salt (pDMAEMA) (Sigma-Aldrich, St. Louis, MO, USA) on the magnetite/silver nanoparticles, the nanoparticles were resuspended using an ultrasonic tip at a frequency of 20 kHz. Next, chloro-silver bonds were formed by exposing the nanoparticles to 1 mM HCl solution until the solution reached a pH 3. The reaction proceeded under vigorous constant stirring at 500 RPM for 10 min. The nanoparticles were then washed with abundant Milli-Q water to remove the excess of hydrochloric until the pH of the suspension was above 6. In order to obtain the magnetite/silver-pDMAEMA conjugates, the pDMAEMA was finally conjugated by a Hofmann elimination reaction with the chlorine groups by adding 10 mg/mL of the polymer solution under constant mechanical stirring at 500 RPM and 50 °C for 1 h.

### 2.3. Conjugation of Buforin II (BUF-II) on Magnetite/Silver Nanoparticles

**Figure 2:**
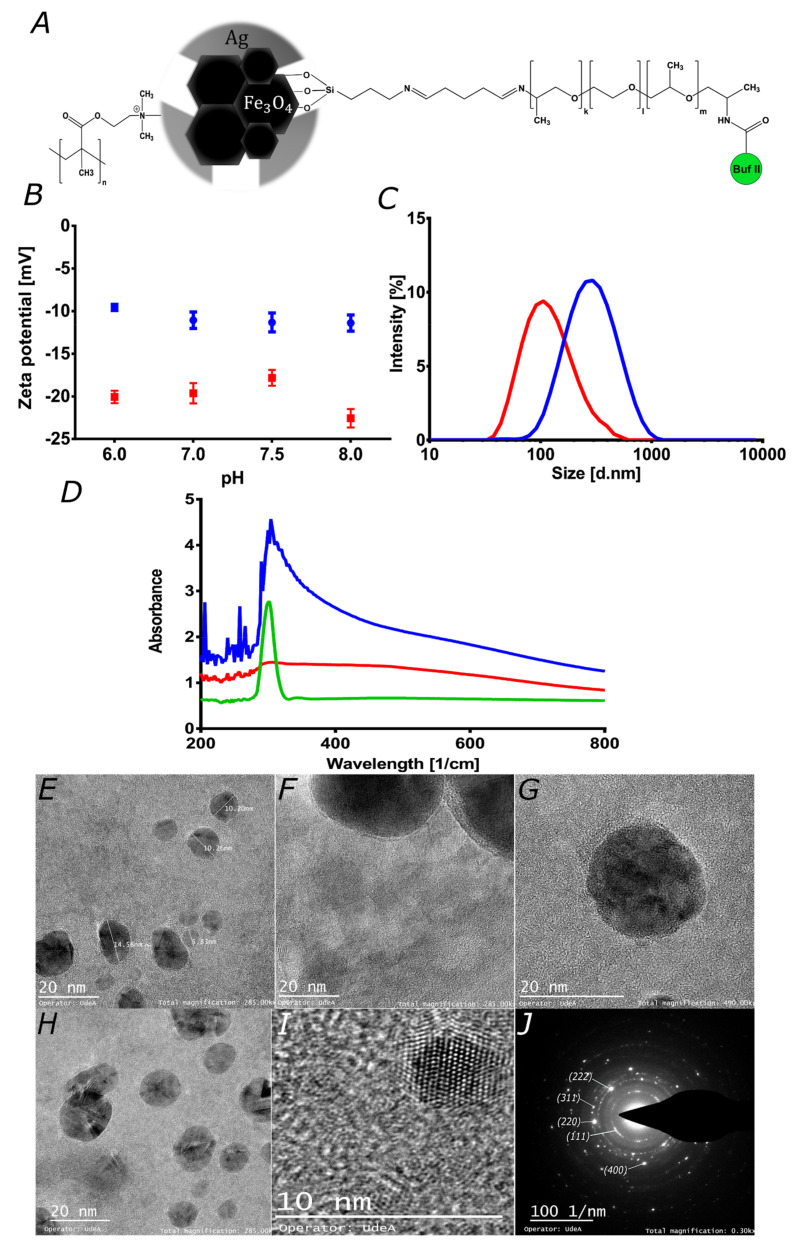
Characterization of magnetite and magnetite/silver. (**A**) Schematic of the chemical structure for the proposed magnetite/silver-pDMAEMA-PEA-BUF-II nanobioconjugate. (**B**) Zeta potential of magnetite (red) and magnetite/silver (blue) nanoparticles. (**C**) Nanoparticles size distribution via Dynamic Light Scattering for magnetite (red) and magnetite/silver (blue). The size of individual particles was visualized in TEM images. (**D**) UV-Visible spectra of magnetite (red), silver NPs (green) and magnetite/silver (blue). These spectra were collected to verify the coverage of silver on magnetite nanoparticles. TEM images of (**E**) magnetite/silver nanoparticles with diameters between 6.8 and 14.5 nm, and (**F**) magnetite clusters covered by a thin layer of silver. (**G**) There seems to be an uneven silver distribution, as evidenced by the coverage surrounding the magnetite clusters shown in the image. (**H**) Clusters of magnetite covered by silver, which is likely to alter the porosity of as-synthesized magnetite, where it was possible to identify different interplanar distances that correspond to iron oxide and silver crystals. (**I**) Interplanar distance confirms the presence of iron oxide and silver. (**J**) SAED pattern of magnetite/silver nanoconjugates confirms interplanar distances measured in H.

In order to obtain the magnetite/silver-pDMAEMA-PEA-BUF-II nanobioconjugates shown in Figure 2A, the magnetite/silver-pDMAEMA conjugates were initially functionalized with 200 μL of (3-aminopropyl) triethoxysilane (APTES, 98%, Sigma-Aldrich, St. Louis, MO, USA) and sonication with a sonic tip for 30 min. This silanization reaction was conducted on the accessible magnetite core, where the silver coverage was incomplete. The purpose of this step was to render free pendant amine groups for the subsequent conjugation of the polyether amine (PEA), Jeffamine^®^ ED-600 (Sigma-Aldrich, St. Louis, MO, USA). This homobifunctional surface spacer exhibits two-terminal amine groups as well. Consequently, conjugation proceeded by forming imine groups between the amine groups on the surface of the nanoparticles and one of the terminals of PEA. This reaction was mediated by the linker glutaraldehyde (25% (v/v), Sigma-Aldrich, St. Louis, MO, USA). Briefly, 2 % (v/v) of well-suspended magnetite/silver-pDMAEMA conjugates (2.5 mg/mL) were mixed with 2 mL of glutaraldehyde and left to react for 1 h under magnetic stirring. Then, 100 μL of PEA was added, and the mixture was incubated in an orbital shaker for 24 h at 250 RPM to obtain the magnetite/silver-pDMAEMA-PEA conjugates. Finally, the C-terminal of Buforin II (BUF-II: TRSSRAGLQFPVGRVHRLLRK, synthesized by GL Biochem Shanghai, Shanghai, China) was conjugated to the free amine terminal of PEA by forming an amide bond. This was accomplished by adding 7 mg of *N*-hydroxy succinimide (NHS, 98%, Sigma-Aldrich, St. Louis, MO, USA) and 14 mg of *N*-[3-dimethylammino)-propyl]-*N*’-ethyl carbodiimide hydrochloride (EDC) (98%, Sigma-Aldrich, St. Louis, MO, USA) to a solution of 0.5 mg of BUF-II in 20 mL of MilliQ water. The obtained solution was maintained at 37 °C for 5 min. The magnetite/silver-pDMAEMA-PEA conjugates were then added and sonicated in an ultrasonic bath (Branson, Danbury, CT, USA) for 10 min. The reaction proceeded at room temperature in an orbital shaker for 24 h at 250 RPM.

### 2.4. Labeling of Magnetite/Silver-pDMAEMA-PEA-BUF-II Nanobioconjugates with Rhodamine B

The nanobioconjugates were labeled with rhodamine B for their observation under the confocal microscope. This was achieved by forming amide bonds between the carboxyl groups of rhodamine B and the free amine groups of conjugated BUF-II. Briefly, 30 mg of rhodamine B (95%, Sigma-Aldrich, St. Louis, MO, USA), 12.3 mg of *N*-[3-dimethylammino)-propyl]-*N*’-ethyl carbodiimide hydrochloride (EDC, 98%, Sigma-Aldrich, St. Louis, MO, USA) and 7.4 mg of *N*-hydroxy succinimide (NHS, 98%, Sigma-Aldrich, St. Louis, MO, USA) were dissolved in 2 mL of *NN*-dimethylformamide (DMF, 99.8%, Sigma-Aldrich, St. Louis, MO, USA) and diluted in 3 mL of type I water. The resulting solution was heated to 37 °C under continuous magnetic stirring for 15 min to activate the carboxyl groups for conjugation. Then, the pre-activated rhodamine B solution was added to 40 mL of an aqueous suspension of magnetite/silver-pDMAEMA-PEA-BUFII in type I water (2.5 mg/mL). This was followed by sonication for 5 min (frequency 40 kHz, amplitude 38%). The reaction mixture was left under constant mechanical stirring (200 RPM) at room temperature and complete darkness (to avoid photobleaching) for 24 h. The obtained magnetite/silver-pDMAEMA-PEA-BUF-II-RhodB nanobioconjugates were then washed 10 times with NaCl solution (1.5% (*w*/*v*)), twice with type I water, and finally suspended in 30 mL of type-I water. Finally, the nanobioconjugates were sonicated for 10 min (frequency 40 kHz, amplitude 38%) and stored at 4 °C under complete darkness until further use.

### 2.5. Synthesis of Liposomes and Magnetoliposomes

Liposomes were synthesized by the hydration of the lipid bilayer method [36]. For this, 100 mg of soy lecithin (Sigma-Aldrich, St. Louis, MO, USA) was dissolved in 10 mL of chloroform. The resulting solution was left in a rotary evaporator (Hei-VAP Core, Heidolph, Schwabach, Germany) at 45 °C, 150 RPM, and vacuum for 1 h. Then, 20 mL of PBS (1X) were added and left in the rotary evaporator for one h (55 °C, 150 RPM, and no vacuum). Lastly, the liposomes solution was collected and filtered three times with a 0.22 μm filter (Sartorius, Goettingen, Germany). Liposomes were stored at 4 °C until further use. The magnetoliposomes were synthesized by mixing magnetite/silver-pDMAEMA-PEA-BUFII nanobioconjugates suspended in Dulbecco’s Modified Eagle Medium (DMEM, Gibco, Dublin, Ireland) at 50 μg/mL with 0.1 mg/mL liposomes in PBS (1X) at a 1:1 volume ratio. The synthesized liposomes were characterized with the aid of DLS in a Zeta-Sizer Nano-ZS (Malvern Panalytical, Malvern, UK) and (TEM) Tecnai F30 (FEI Company, Fremont, CA, USA) at a resolution of 134 eV and with reference energy of 5.9 keV.

### 2.6. Characterization of Magnetite/Silver and Surface Conjugations

The hydrodynamic radii and Zeta-potentials of magnetite and magnetite/silver nanoparticles were determined via dynamic light scattering (DLS) in a Zeta-Sizer Nano-ZS (Malvern Panalytical, Malvern, UK). Nanoparticles were diluted in Milli-Q water to form a 1% (*w*/*v*) solution as recommended by the manufacturer and sonicated in an ultrasonic bath to avoid aggregation. Parameters such as refractive index and absorbance were taken into account according to the manufacturer recommendations for nanomaterials. The presence of the silver shell was verified by collecting the absorbance spectrum from 200 cm^−1^ to 1000 cm^−1^ of a suspension of the nanoparticles in type I water (0.1 mg/mL) aided by a UV-Vis spectrophotometer (GENESYS, Thermo Scientific, Waltham, MA, USA). This takes advantage of the absorption of nanosilver in the visible range of the spectrum [29,37]. Conjugation of pDMAEMA and BUFII was corroborated via Fourier transform infrared spectroscopy (FTIR) in a Bruker Alpha II FTIR Eco-ATR instrument (Bruker, Billerica, MA, USA). Spectra were collected from 800 to 4000 cm^−1^ by averaging three scans, smoothing by 13 points, and normalization algorithm. The conjugation efficiency of pDMAEMA and BUFII, as well as the thermal stability of the nanobioconjugates, were estimated via thermogravimetric analysis (TGA) in a simultaneous TGA/DSC instrument (TA Instruments, New Castle, DE, USA). The analysis was carried out by running a linear temperature ramp at a rate of 10 °C/min from 25 to 800 °C under an N_2_ atmosphere. The percentage of weight loss was correlated to the detachment of the different compounds present on the surface nanoparticles. Magnetite/silver and magnetite/silver-pDMAEMA-DNA were imaged using a scanning electron microscope equipped with energy dispersive spectroscopy (SEM+EDS, TESCAN LYRA3 FIB-SEM). Magnetite/silver coverage was analyzed via transmission electron microscopy in a (TEM) Tecnai F30 (FEI Company, Fremont, CA, USA) instrument at a resolution of 134 eV and with reference energy of 5.9 keV.

### 2.7. Loading and Delivery of Circular DNA

Loading and delivery were tested with circular DNA using a typical CRISPR/Cas9 plasmid with a total size of 10,249 bp. The vector contains the information encoding for the Cas9 enzyme, guide RNA (gRNA), a mCherry fluorescent reporter, and the ampicillin resistance gene for selection. The plasmid was produced by growing *Escherichia coli* BL21 overnight in LB liquid media. Plasmid DNA was extracted using the QIAprep Spin Miniprep kit (QIAGEN, Germantown, MD, USA) in a Tris-Acetate-EDTA (TAE) at pH 8.5 by following the manufacturer instructions. The DNA loading and delivery were tested in vitro by suspending 250 μg of nanoparticles in Phosphate-buffered saline (PBS) buffer at pH 6. This was followed by precipitating and adding the DNA solution to complete the loading. For the delivery, the loaded nanoparticles were washed twice with TAE buffer at pH 8.5, then precipitated and exposed to a PBS buffer at pH 6. DNA was quantified using a nanodrop spectrophotometer (DeNovix DS—11 Microvolume UV-Vis Spectrophotometer). Loaded DNA was calculated by subtracting the DNA remaining in solution from the total DNA added to the magnetite/silver-pDMAEMA conjugate. Loading efficiency was determined as the DNA loaded to the magnetite/silver-pDMAEMA conjugate divided by total added DNA (see Equation (1)). The instantaneous delivery efficiency was calculated as the total DNA delivered divided by the added DNA (see Equation (2)). The loading capacity was determined as the maximum amount of DNA loaded per weight of nanoparticles. The experiment was conducted by adding DNA stepwise to 50 μg of the magnetite/silver-pDMAEMA conjugate until saturation was reached.

(1) $$\eta_{loading}\;=\;\frac{DNA_{load}}{DNA_{add}}\times 100$$

(2) $$\eta_{delivery}=\frac{DNA_{deliver}}{DNA_{add}}\times 100$$

### 2.8. Hemolysis Assay

Hemolytic activity of the magnetite/silver, magnetite/silver-pDMAEMA, and magnetite/silver-pDMAEMA-PEA-BUF-II nanobioconjugates, liposomes and magnetoliposomes was tested on erythrocytes isolated from freshly drawn blood of a healthy human donor. The collected blood was centrifuged at 1800 RPM for 5 min, and the supernatant with the plasma was discarded. The precipitate containing the erythrocytes was resuspended and subsequently washed five times with NaCl solution (0.9 % (*w*/*v*)) and once with PBS (1X). An erythrocytes stock was then prepared by adding 1 mL of isolated erythrocytes (4.3 × 10^6^ erythrocytes/μL) in 9 mL of PBS (1X). Serial dilutions of the nanobioconjugates from 200 to 12.5 μg/mL were prepared by mixing initial stocks with PBS (1X). Liposomes and magnetoliposomes were tested at 0.1 mg/mL. Triton X-100 (1% (*v*/*v*)) and PBS (1X) were used as positive and negative controls, respectively. One-hundred microliters of each treatment were seeded with 100 μL of erythrocytes stock and incubated at 37 °C, 5% CO_2_ for 1 h. Samples were centrifuged at 1800 RPM for 5 min. Finally, 100 μL of each supernatant was placed in a 96-well microplate and read at 450 nm in a microplate reader. Hemolysis percentage was calculated by subtracting the absorbance of the negative control from the test sample and dividing by the difference of the controls (positive control—negative control).

### 2.9. Platelet Aggregation Assay

Platelet aggregation tendency of the magnetite/silver; magnetite/silver-pDMAEMA; and magnetite/silver-pDMAEMA-PEA-BUF-II nanobioconjugates, liposomes, and magnetoliposomes was tested on platelets isolated from freshly drawn blood of a healthy human donor. The blood sample was collected in a vacutainer tube with sodium citrate as an anticoagulant to avoid platelet aggregation. The sample was centrifuged at 1000 RPM for 15 min at room temperature to obtain the platelet-rich plasma (PRP). Serial dilutions of the nanobioconjugates from 200 to 12.5 μg/mL were prepared by mixing initial stocks with PBS (1X)). Liposomes and magnetoliposomes were tested at 0.1 mg/mL. Thrombin (6U) was used as a positive control, while PBS (1X) served as a negative reference. Fifty microliters of PRP were seeded with 50 μL of the treatments in a 96-well microplate and incubated at 37 °C, 5% CO_2_ for 5 min. Finally, 50 μL of each supernatant was extracted and transferred on a 96-well microplate. Absorbance was read at 620 nm in a microplate reader.

### 2.10. LDH Cytotoxicity Assay

Cytotoxicity of the magnetite/silver; magnetite/silver-pDMAEMA; and magnetite/silver-pDMAEMA-PEA-BUF-II nanobioconjugates, liposomes and magnetoliposomes was carried out by quantification of the lactate dehydrogenase enzyme (LDH), using a commercially available Cytotoxicity Detection Kit (LDH) (Roche, Basel, Switzerland). Cytotoxicity was tested by exposing the samples to Vero cells (ATCC^®^ CCL-81) at a cell density of 100,000 cells/mL. Serial dilutions (i.e., 200–12.5 μg/mL) were prepared by mixing concentrated stocks with DMEM media. Also, liposomes and magnetoliposomes were tested at 0.1 mg/mL. Triton X-100 (1% (v/v)) was used as positive control and DMEM media as the negative one. One-hundred microliters of the cell stock (DMEM, supplemented with 10% (v/v) FBS) were deposited in 96-well microplates (10,000 cells/well) and incubated at 37 °C, 5% CO_2_ for 24 h. After incubation, culture media was removed, and cells were washed with PBS (1X). Next, PBS was removed, and 100 μL of the different samples were added and incubated at 37 °C, 5% CO_2_ for 24 and 48 h. Finally, supernatants (50 μL) were transferred to 96-well microplates along *with* 50 μL of the reaction mixture (Cytotoxicity Detection Kit (LDH), Roche, Basel, Switzerland) and left to react under mechanical stirring (50 rpm), room temperature and complete darkness for 15 min. Absorbance was finally read at 490 nm in a microplate reader.

### 2.11. Cell Translocation and Endosome Escape

The endosomal escape of magnetite/silver-pDMAEMA-PEA-BUF-II-RhodB nanobioconjugates was assessed by colocalization between them and Lysotracker Green DND-26 (Thermo Fisher, Waltham, MA, USA) after internalization into Vero (ATCC^®^ CCL-81) and neuroblastoma (ATCC^®^ SH-SY5Y) cells. Fifty-thousand cells per well were seeded on a glass slide that was previously placed in a 24-well microplate. The cells were incubated in DMEM medium supplemented with 10% (*v*/*v*) FBS for 24 h (37 °C, 5% CO_2_) to allow cell adhesion. After incubation, the cells were exposed to labeled nanobioconjugates for 0.5 h and 4 h. Then, the cells were washed three times with DMEM medium and exposed to DMEM solution with Hoechst 33342 (Thermo Fisher, Waltham, MA, USA) (1: 1000) and Lysotracker Green DND-26 (1: 10,000) for 10 min before observation via confocal microscopy. The images were obtained using an Olympus FV1000 confocal laser scanning microscope (CLSM) with a PlanApo 60x oil immersion objective. Excitation/Emission wavelengths of 358 nm/461 nm, 488 nm/520 nm, and 546 nm/575 nm were used for the detection of nuclei, endosomes, and nanoparticles, respectively. Ten images were taken for each treatment (10 cells per image). The internalization of the nanobioconjugates and the cytosol distribution were studied by calculating the surface area coverage. Images analysis was performed with the aid of Fiji-ImageJ software [38]. Data analysis was completed using the GraphPad Prism V 6.01 software (GraphPad Software, La Jolla, CA, USA). Statistical comparisons were made using the unpaired t-test. Results of *p* ≤ 0.05 (*) were considered significant. Data are given as average ± one standard deviation.

## 3. Results

Magnetite was synthesized by co-precipitation, which has been reported as one of the simplest synthesis methods [39,40]. Even though the particle size distribution obtained is ampler than with other methods [41], co-precipitation is an attractive route from the scaling-up viewpoint as no expensive reagents or extreme process conditions are required. Additionally, the silver shell was synthesized by a “green synthesis” scheme with commercially available honey as a reducing agent [37]. Finally, the surface spacer (PEA) and pH-responsive (pDMAEMA) polymers can be easily purchased at relatively low costs. The scope of the study is to deliver plasmids responsively. To achieve this, we considered the use of pDMAEMA, which had previously demonstrated pH-responsiveness at the pH values commonly found in the cytoplasm [20,42]. The most straightforward route for conjugation of pDMAEMA was via chlorine chemistry. However, that is elusive on the magnetite surface but is very conventional on gold or silver [34,37]. Due to our experience with silver, we decided to synthesize a patchy shell of it on magnetite, so we could still use our previous developments to achieve high cell penetration levels and endosomal escape with the aid of the antimicrobial peptide Buforin II [35,43]. Also, a liposomes entrapment was used to potentiate the nanoconjugate’s stability.

### 3.1. Characterization Magnetite/Silver Nanoparticles

Zeta-potentials of the magnetite and magnetite/silver nanoparticles were measured at different pH values to determine their colloidal stability under different environmental conditions. This is of considerable importance for the development of stable nanovehicles for drug delivery [44]. Figure 2B shows the obtained Zeta-potentials in the pH range from 6 to 8. In all cases, the Zeta-potential remains negative, which may be useful to improve nanomaterial cellular uptake [25,41]. Also, it shows how both magnetite and magnetite/silver exhibit similar behavior. However, in the case of magnetite/silver, there is a considerable decrease in the absolute value of the Zeta potential, thereby making them less colloidally-stable [44]. This could be related to the electrical properties and shape of silver [45,46]. The magnetite and magnetite/silver nanoparticles show hydrodynamic diameters larger than those observed in the TEM images, as shown in Figure 2C–E. This could be related to the aggregation of the nanoparticles and their low colloidal stability [30,44]. To avoid aggregation of the nanoparticles, it is possible to use mechanical methods such as sonication or chemical methods such as a suspension in ionic solvents [30]. Here, we avoided aggregation by sonicating the nanoconjugates in an ultrasonic bath. This procedure was conducted before each conjugation step and prior to characterization. The presence of the silver shell was determined spectrophotometrically by collecting the absorbance in the 200–800 cm^−1^ range [28,29]. Figure 2D shows that for the magnetite/silver nanoparticles, the absorbance between 300 and 400 cm^−1^ increased concerning bare magnetite. Albeit at a higher level, this absorbance is comparable with that reported for pure silver nanoparticles, thereby validating the presence of silver superficially [28,29,37]. Both the Z-potential and UV-Vis suggested an incomplete or discontinuous coating of the silver shell. First, the Z-potential of core/shell nanoparticles increased concerning that of magnetite; however, the reported values for silver nanomaterials are lower. This likely indicates a discontinuous silver coverage. Further evidence was provided by the UV-Vis spectrum, which shows the characteristic peak of silver but also that of magnetite.

TEM imaging was conducted to investigate the size, size distribution, clustering, morphology, and coverage of the silver shell. Figure 2E–G show the TEM images for the magnetite and magnetite/silver nanoparticles. According to the images, the average size of individual magnetite particles approaches 10.4 nm, which agrees well with sizes reported elsewhere for bare magnetite synthesized via coprecipitation [43,47]. For the magnetite/silver nanoparticles, the average diameter increased by about 4 to 8 nm, which has also been reported for similar systems [28]. Figure 2F–G confirm that the silver coating of the magnetite core is unevenly distributed on the surface and is formed around clusters. These results agree well with those of Garza–Navarro et al., who also found a heterogeneous coverage of silver [48]. Figure 2I shows interplanar distances of 2.45, 2.12, and 2.87 Å, which correspond to the crystalline structure of magnetite [48]. Also, at the edges of the covered clusters, the found interplanar distance decreases to 1.45 Å, which has been reported for silver [48]. Taken all together, the UV-Vis absorbance, SEM, and TEM images confirmed that magnetite/silver nanoparticles were synthesized adequately with morphologies and sizes similar to those reported elsewhere. The unevenness of the silver coverage was exploited to successfully conjugate Buforin II with the aid of a polyether amine extensor by following the procedures developed by us previously [35].

### 3.2. Characterization of Liposomes and Magnetoliposomes

**Figure 3:**
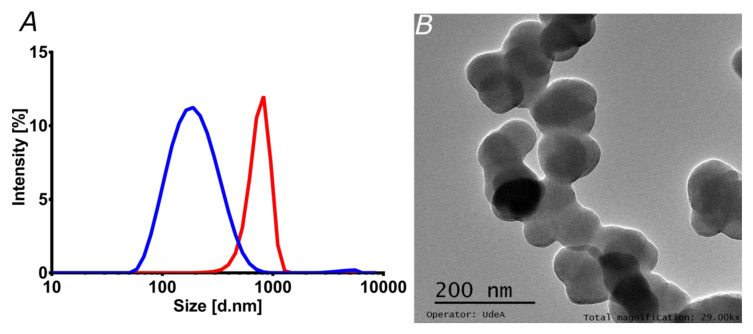
Characterization of liposomes and magnetoliposomes. (**A**) Liposomes (blue) and magnetoliposomes (red) size distribution by Dynamic Light Scattering. The size was estimated using the refractive index and absorbance parameters of the phospholipid membrane. (**B**) TEM image of the liposomes synthesized by the hydration of the lipid bilayer method.

Liposomes have been used in the loading, transport, and delivery of different pharmacological molecules and nanocarriers [24,36]. Here, the encapsulation in liposomes had two main intentions. On the one hand, increasing stability might eventually prolong in vivo circulation time, which, in turn, is critical for a long-lasting therapeutic effect [49]. On the other hand, enhanced penetration potency might be reflected in superior bioavailability, as has been shown previously in similar studies [24,36]. This may ultimately lead to a reduced intake for the patients [2,49]. Figure 3A shows the particle size distribution of liposomes and magnetoliposomes, as estimated by DLS. The average hydrodynamic diameter of the liposomes approached 200 nm, whereas the one for magnetoliposomes reached 750 nm. This was corroborated via TEM (Figure 3B), where the estimated size of individual liposomes was in the range of 72.5 to 92.0 nm, with an average size of 84.2 nm. The estimated sizes agree well with those reported elsewhere [24].

### 3.3. Characterization of Nanoconjugates and Nanobioconjugates

**Figure 4:**
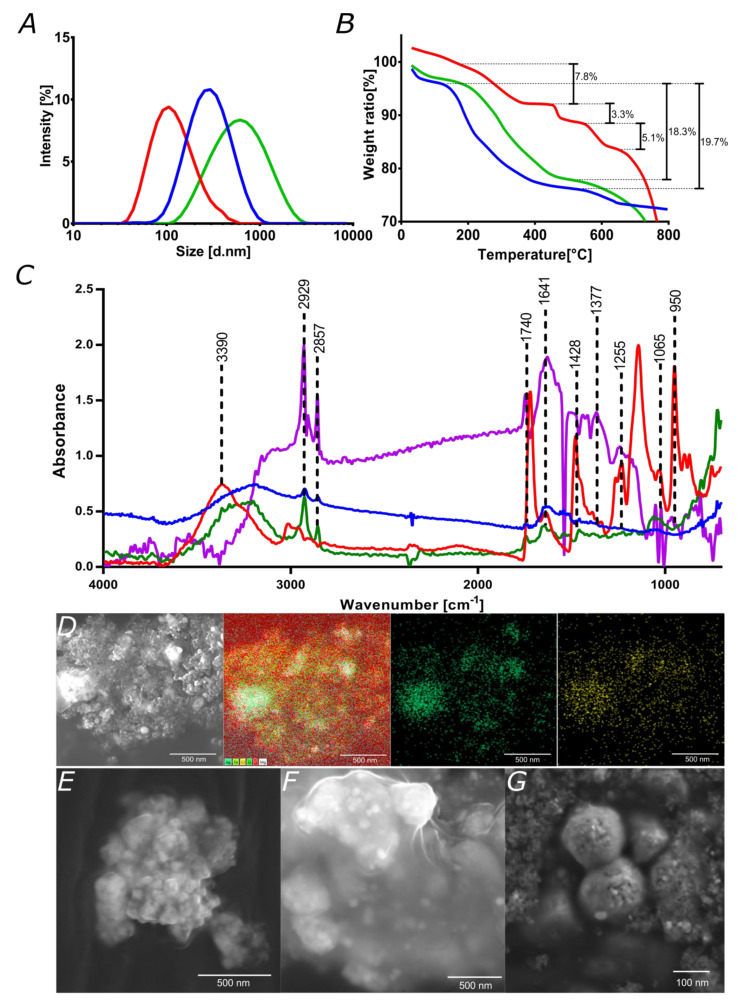
Characterization of nanoconjugates and nanobioconjugates. (**A**) Nanoparticles size distribution using Dynamic Light Scattering for magnetite (red), magnetite/silver (blue), and magnetite/silver-pDMAEMA-PEA-BUFII (green). (**B**) TGA analyses of magnetite (green), magnetite/silver (blue), and magnetite/silver-pDMAEMA-PEA-BUFII (red); percentage of weight loss are presented in each case. (**C**) FTIR spectra of bare pDMAEMA (Red), magnetite/silver (Blue), magnetite/silver-pDMAEMA (green) and magnetite/silver-pDMAEMA-PEA-BUFII (purple). Peaks at 1740 and 1428 cm^−1^ are for C=O and -N(CH_3_)_2_ bonds of conjugated pDMAEMA while peaks at 1641, 1377, and 1255 cm^−1^ correspond to amide I and α helix, stretching vibration of the N-C bond, and the amide II, respectively, of the conjugates BUF-II. Peaks 1065 cm^−1^ and 950 cm^−1^ are for the Si–O–Si (due to silanization) and Fe–O bonds of the magnetite core. (**D**) SEM image of magnetite/silver nanoparticles. The silver shell and the success of the chlorination reaction were verified through EDS analysis. The EDS map shows silver in green and chlorine in yellow, which superimposed and confirmed that chlorination was completed to a large extent. Scale bar corresponds to 500 nm. (**E**) SEM image of the core magnetite nanoparticles as synthesized by co-precipitation (Scale bar corresponds to 500 nm). (**F**) SEM image of magnetite/silver-pDMAEMA shows the effective coating of nanoparticles by the polymer. Scale bar corresponds to 500 nm. (**G**) Magnetite/silver-pDMAEMA-DNA after loading in buffer at pH 7 (scale bar corresponds to 100 nm).

Hydrodynamic diameters and thermogravimetric analyses of magnetite, magnetite/silver, and magnetite/silver-pDMAEMA-PEA-BUFII are presented in Figure 4A,B respectively. Bare magnetite nanoparticles exhibited a mean hydrodynamic diameter of 124 nm, which increased to 342 nm after forming the silver shell. The corresponding polydispersity indexes (PI) decreased from 43.5% to 22.6%, which are comparable with those reported elsewhere [48]. The magnetite/silver-pDMAEMA-PEA-BUFII nanobioconjugates exhibited a hydrodynamic diameter of 462 nm, which can be attributed to the presence of the PEA extensor needed for the conjugation of Buforin II (Figure 4A). The obtained hydrodynamic diameter is comparable to that observed previously by us after conjugation of Buforin II on PEA-modified magnetite nanoparticles [35,43]. The sizes are in the range of those acceptable for in vitro and in vivo applications, as they can still permeate cells without inducing significant cytotoxicity, hemolytic, or platelet aggregation effects (see biocompatibility and penetration results below).

Thermal stability analyses were conducted with the aid of TGA to estimate the completeness of the different synthesis steps and the conjugation efficiencies. Figure 4B shows that magnetite and magnetite/silver nanoparticles exhibit a weight loss before 100 °C, most likely due to moisture adsorbed on the samples. A subsequent loss step above about 200 °C might be associated with excess reagents from the synthesis, which most likely remained absorbed on the nanoparticles. Such losses were 19.7% and 18.3% for magnetite and magnetite/silver, respectively. These results agree well with previous TGA analyses of similar systems [48]. The magnetite/silver-pDMAEMA-PEA BUFII nanobioconjugates showed three larger weight losses. The first one was 7.8% and occurred above about 200 °C, which can be correlated with excess reagents. The reduced loss compared with those of magnetite and magnetite/silver is most likely due to a more thorough washing scheme for the nanobioconjugates. The second loss step of 3.3% was above 440 °C and can be related to the decomposition of pDMAEMA and PEA, which is comparable with that reported elsewhere [50]. The last weight loss step of 5.1% took place above 540 °C and most likely corresponds to the detachment of conjugated Buforin II. Albeit at a lower level, this conjugation efficiency is close to that determined by us previously in closely related systems [35,43].

FTIR analyses were also conducted to verify the presence of conjugated molecules on the surface of the nanoparticles. Figure 4C compares the spectra for the magnetite/silver-pDMAEMA-PEA-BUFII nanobioconjugates with those of magnetite/silver nanoparticles, pDMAEMA, and magnetite/silver–pDMAEMA. The presence of the C–C and C–H stretching vibration in the spectrum of the nanobioconjugates at 3390 cm^−1^ present in the pDMAEMA spectra [26,51]. This is also the case for the peaks at 2857 cm^−1^ and 2929 cm^−1^, which correspond to the main chain of C–C and C–H stretching vibrations, respectively [26,51]. Moreover, the stretching vibration at 1740 cm^−1^ corresponds to the carbonyl C=O group while that at around 1428 cm^−1^ is for the deformational stretching vibration of the –N(CH_3_)_2_ group of pDMAEMA. The obtained results agree well with those reported for the same polymer elsewhere [26,51]. Conjugation of Buforin II was verified by the presence of 1641, 1377, and 1255 cm^−1^ peaks, which are related to the amide I and α helix of the peptide, stretching vibration of the N–C bond, and the amide II band, respectively [43,52]. The peaks at 1065 cm^−1^ and 950 cm^−1^ are for the Si–O–Si (due to silanization) and Fe–O bonds of the magnetite core, respectively [39].

Finally, SEM imaging allowed checking the presence of the pDMAEMA on the surface after conjugation to the chlorinated magnetite/silver nanoparticles. Also, the loading of DNA was observed via SEM. The presence of silver and the extent of chlorination were qualitatively assessed with the aid of the EDS detector of the SEM instrument. This provided further evidence of the uneven coverage of silver. The chlorine signal colocalized on that of silver, thereby demonstrating the success of chlorination, which was critical for the subsequent covalent attachment of the pDMAEMA through a Hoffman elimination reaction [23,26]. Figure 4F shows the magnetite/silver nanoparticles after conjugation of pDMAEMA. Images suggest that nanoparticles are embedded within a matrix that is likely formed by the coil-coiled interactions of pDMAEMA in a dried state. The characterization results provided evidence for the effective synthesis and functionalization of the designed nanobioconjugates. Finally, Figure 4G demonstrated qualitatively that after exposing the magnetite/silver-pDMAEMA nanoconjugates to a typical CRISPR-Cas 9 circular vector, they undergo an observable change in size most likely due to the complexation of DNA by the polymer.

### 3.4. DNA Loading and Delivery

**Figure 5:**
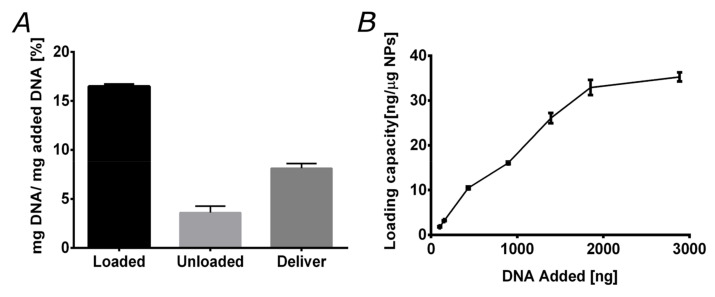
Nanoconjugate loading and delivering capacities. (**A**) Loading and delivery capacities of the magnetite/silver-pDMAEMA nanoconjugates. The loading capacity approaches 16%, while instant delivery capacity is around 8%. (**B**) magnetite/silver-pDMAEMA-PEA-BUFII loading capacity (defined as the ng of DNA loaded per μg of the nanoconjugate) exhibits an initial linear regime for DNA amounts up to 2000 ng followed by saturation above 3000 ng.

Experiments of in vitro loading and delivery of plasmid DNA were conducted with the magnetite/silver-pDMAEMA nanoconjugates to evaluate their efficiency to carry DNA and deliver it under the appropriate pH conditions. Accordingly, the response of the vehicle was considered under varying pH conditions, i.e., in the range from 6.5 to 8, where the polymer undergoes a transition from an extended to a coil-coiled conformation. Here, we took advantage of the ability of pDMAEMA to form polyplexes and subsequently deliver the nucleotide cargoes under intracellular pH conditions. For this reason, pDMAEMA has been extensively used to achieve high transfection rates [20,42]. Figure 5A shows the efficiencies of the vehicle based on the total DNA added, the discarded DNA during washing steps, and that instantaneously released. Loading was conducted at pH 8, where pDMAEMA interacts with DNA in a compact conformation. Our results indicate that the magnetite/silver-pDMAEMA conjugate can load about 16.4% of available DNA in solution. The remaining DNA was lost during the washing steps.

DNA release experiments were performed at pH 6.5, where pDMAEMA relaxes back to an extended conformation. The delivery efficiency was found to be 8.1% of the initially added DNA. We also explored the maximum loading capacity of the magnetite/silver-pDMAEMA-PEA-BUFII nanobioconjugates by varying the initially added DNA. The results are shown in Figure 5B and corroborate that a maximum loading capacity of about 35 ng of DNA per μg of nanobioconjugate was reached for an initial load of 3000 ng (3 μg) of DNA. A similar limit has been reported for closely related nanovehicles for gene delivery [26]. This encouraging result indeed confirms that our nanobioconjugates are well-suited for an important loading amount of DNA.

### 3.5. Biocompatibility

**Figure 6:**
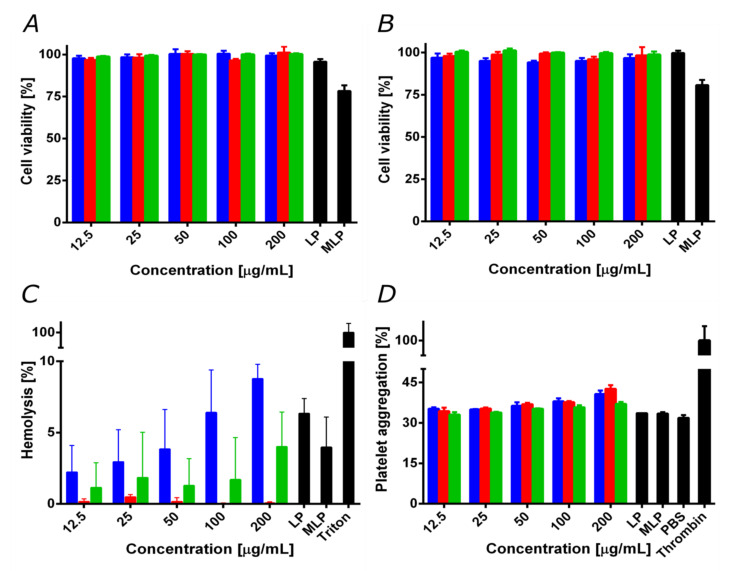
Biocompatibility assays for magnetite/silver (blue), magnetite/silver-pDMAEMA (red) and magnetite/silver-pDMAEMA-PEA-BUFII (green). LP represents free liposomes and MLP magnetoliposomes. (**A**) Cytotoxicity by LDH in Vero cells line after 24 h. (**B**) Cytotoxicity by LDH in Vero cells after 48 h. Except for MLP, cell viability remained above 95%. (**C**) The hemolysis assay shows an average hemolytic effect below 9% in all cases. The positive control was Triton 100-X. (**D**) Platelet aggregation assay shows an aggregation tendency of about 40% for all treatments. The negative control was PBS 1X, while the positive one was thrombin.

For medical applications, it is crucial to evaluate potential biocompatibility issues of new materials. This is important to anticipate possible drawbacks when translating the developments into pre-clinical and clinical applications [3]. Accordingly, we evaluated cytotoxicity, and hemolytic and platelet aggregation tendency. Figure 6A,B show cell viability in Vero and after exposure to the treatments for 24 and 48 h, respectively. The nanoconjugates, nanobioconjugates, and liposomes showed cell viability levels above 95% even after 48 h of incubation. This was not the case of the magnetoliposomes that led to a significant decrease in cell viability to about 80%.

The negligible impact of our nanobioconjugates on cell viability contrast with the reported cytotoxicity and genotoxicity of silver in mammalian cells [53,54], as well as that of pure pDMAEMA [24,55]. In the case of silver, this has been attributed to the marked tendency of silver ions to induce the generation of reactive oxygen species and, consequently, detrimental oxidative stress [54,56]. In the case of pDMAEMA, the tertiary amino groups on its structure have been considered responsible for altering cell membrane stability. These issues have been tackled by increasing the particle size, decreasing the silver content, or modifying the surface with biopolymers or other biocompatible molecules capable of restricting the release of silver ions [56,57,58,59,60,61,62,63,64,65]. Accordingly, the low cytotoxicity of our nanobioconjugates can be explained by the relatively low content of silver, as well as its uneven surface coverage (see discussion above).

Moreover, the PEA and pDMAEMA used for conjugation have been reported to increase their biocompatibility upon chemical modification or conjugation on nanomaterials [27,55,66]. This result agrees with previous reports of cytotoxicity for different core/shell materials [27]. We have recently applied this approach for the immobilization of Buforin II on PEA-modified magnetite nanoparticles to assure high biocompatibility [43].

The decrease in cell viability of the magnetoliposomes is most likely due to the capability of this vehicle to promote the massive internalization of nanobioconjugates, thereby leading to an exacerbated cytotoxic response. This has been reported previously for the delivery of pDMAEMA polyplex with doxorubicin encapsulated in liposomes to HepG-2 cells for the treatment of tumor cells [24]. Despite the reduction in cell viability, this result demonstrated the potency of the magnetoliposomes in penetrating cell membranes, and therefore increasing bioavailability. This will most likely lead to a significant decrease in the dose required to achieve the same therapeutic efficacy.

Since the primary administration strategy of the developed vehicle is most likely to be intravenous (IV), we analyzed the impact on red blood cells and the potential thrombogenic activity of our nanobioconjugates. Figure 6C shows that the average hemolytic activity for the different treatments remained below 9% for concentrations up to 200 μg/mL. Nevertheless, the magnetite/silver nanoparticles exhibited the highest hemolytic activity of all treatments, followed by the magnetite/silver-pDMAEMA-PEA-BUFII nanobioconjugates. These results are consistent with previous reports that showed the high hemolytic effect of the silver [67] and the moderate hemolytic effect of pDMAEMA [62]. Nevertheless, it is essential to note that the hemolysis percentage of the obtained magnetite/silver and magnetite/silver-pDMAEMA-PEA-BUFII nanobioconjugates is still lower than that of the silver nanoparticles and free pDMAEMA. This can also be explained in light of the notion that upon immobilization, these molecules exhibit an increase in biocompatibility [55,61,63]. Finally, Figure 6D shows the results of the interaction of the different treatments with human platelets. The samples showed a similar platelet aggregation percentage (i.e., about 30%) compared to the negative control, PBS 1X. However, a slight increase (of about 5%) in platelet aggregation was observed as the concentration of the treatments was increased above 100 μg/mL. This result agrees with previous reports that established a relation between the amount of silver and pDMAEMA with an increased thrombogenesis and platelet aggregation [62,64]. The obtained results confirm the high biocompatibility of the magnetite/silver-pDMAEMA-PEA-BUFII nanobioconjugates and the magnetoliposomes in terms of hemolysis, platelet aggregation, and LDH cytotoxicity.

### 3.6. Cell Translocation and Endosome Escape

**Figure 7:**
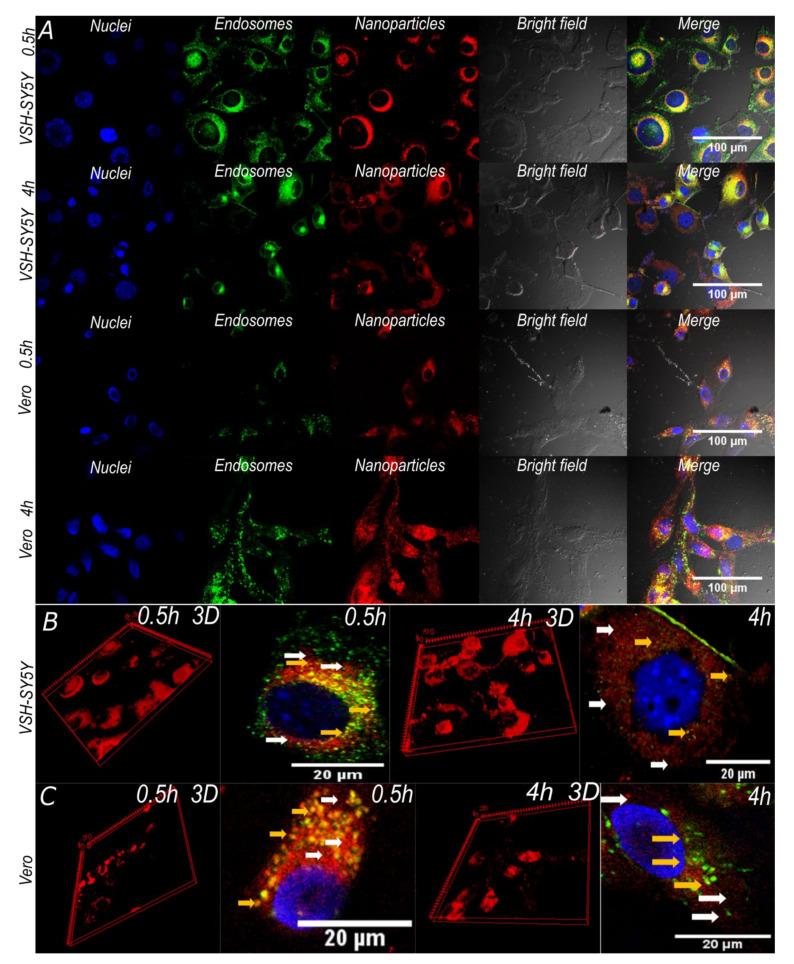
Confocal images of SH-SY5Y and Vero cells exposed to magnetite/silver-pDMAEMA-PEA-BUFII-RhodB nanobioconjugates for 0.5 and 4 h. (**A**) The first two channels correspond to nuclei (blue) and endosomes (green) labeled with Hoechst 33342 and Lysotracker green DND-26, respectively. The third channel corresponds to nanobioconjugates labeled with rhodamine-B (red) and the fourth to the DIC field that shows cell morphology and integrity. Finally, the merge of all channels’ highlights nanoparticle cellular internalization (yellow areas correspond to colocalization between red and green channels). (**B**, **C**) SH-SY5Y and Vero cells exposed to magnetite/silver-pDMAEMA-PEA-BUFII-RhodB nanobioconjugates for 0.5 h and 4 h. The 3D reconstructions show the distribution of the nanobioconjugates throughout the cells’ cytoplasm. The white arrows point to endosomal escape areas (red areas), while the yellow ones showed colocalization of the nanobioconjugates and endosomes where no endosomal escape was achieved (yellow areas).

Cell integrity is maintained after incubation with the nanoconjugates and nanobioconjugates for 0.5 and 4 h. This is observed in Figure 7A, where the cells showed unfragmented nuclei, no cell membrane shrinkage, and absence of apoptotic bodies and cell rounding. This indicated that the analyzed cells were healthy and showed no signs of entering into apoptotic processes [65,66]. These results were corroborated by the cytotoxicity analysis discussed above, where cells exposed to nanoparticles for 48 h maintained high viability. Additionally, the 3D cell reconstructions (Figure 7B,C) allowed us to verify that the magnetite/silver-pDMAEMA-PEA-BUFII-RhodB nanobioconjugates were effectively internalized and distributed throughout the entire cytoplasm without noticeable agglomeration [68].

**Figure 8:**
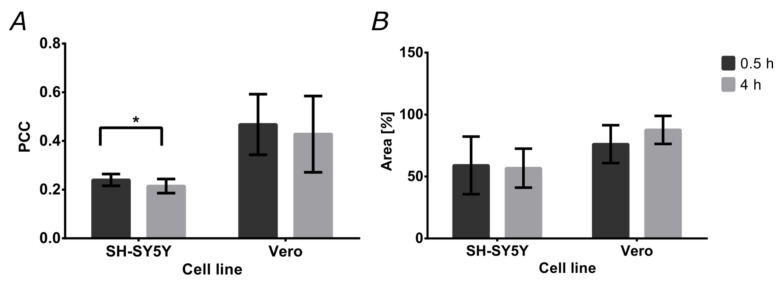
Percentages of colocalization and cytoplasm area covered by the nanobioconjugates. The analysis was conducted on confocal microscopy images. (**A**) Pearson’s correlation coefficient (PCC) for both cell lines after the two exposure times (0.5 and 4 h). (**B**) Cytoplasm area coverage by the nanobioconjugates for both cell lines after the two exposure times (0.5 and 4 h).

Colocalization of the magnetite/silver-pDMAEMA-PEA-BUFII-RhodB nanobioconjugates with Lysotracker Green is an indication of failure to escape endosomes. This was quantitatively analyzed with the aid of the Pearson’s correlation coefficient (PCC), which varies from 0 to 1 for no colocalization to complete colocalization. As a result, a PCC of “0” indicates complete endosomal escape, while “1” is for the absence of any endosomal escape. Figure 8A shows the PCCs for SH-SY5Y and Vero cells after incubation with the nanobioconjugates for 0.5 and 4 h. In the case of SH-SY5Y cells, the PCC approached 0.240 ± 0.024 after 0.5 h and decreased to 0.215 ± 0.029 in a statistically significant manner after 4 h. This indicates an increase in the tendency of the nanobioconjugates to escape endosomes. For Vero cells, colocalization remained around 0.4 (0.468 ± 0.124 after 0.5 h, 0.428 ± 0.157 after 4 h). This suggests that after initial penetration and escape, the nanobioconjugates are not able to continue escaping endosomes. Figure 8B shows the cell coverage by the nanobioconjugates for the two cell lines after 0.5 and 4 h. For the SH-SY5Y cells, coverage of the cytoplasmic area was maintained close to 50% in both cases (59.01% ± 23.24% after 0.5 h, and 56.74% ± 15.73% after 4 h), which confirms the internalization of the nanobioconjugates and their stability throughout analyzed the time. These results are corroborated with those obtained in Vero cells where the percentage of cytoplasmatic area covered by the nanobioconjugates after 0.5 h was 76.12% ± 15.31% and 87.63% ± 11.32% after 4 h.

The complete cell internalization of our nanobioconjugates appears to be a rapid process as compared with similar reports that indicate that, in some cases, the process can last for 8 h [69]. The ease of translocation observed for our nanobioconjugates might be related to a facilitated penetration pathway that is enabled by an altered conformation of the immobilized polymers (i.e., PEA and pDMAEMA). In this regard, it has been reported that upon immobilization, some polyelectrolytes tend to have an increased strength [31] and charge reorganization that promotes intermingling with membrane phospholipids that eventually results in translocation [70]. This is, in fact, the case of pDMAEMA immobilized on the surface of the nanoparticles, which has proven abilities to adsorb and penetrate cell membranes [26,71,72]. Furthermore, Buforin II immobilization on the surface facilitates their internalization, since this cationic peptide interacts with membrane proteins, triggering membrane translocation processes [35,73].

The entrapment of nanobioconjugates in endosomes strongly indicates that some of the vehicles might be entering the cells by clathrin-mediated endocytosis [74]. This is also related to the presence of corona proteins around the nanobioconjugates as a consequence of interactions with the serum present in the cell culture medium [69]. Additionally, some of the free cytoplasm nanoconjugates likely entered the cells by a multi-pathway mechanism that involves either clathrin-mediated or caveolin-mediated common pathways used for endocytosis of larger molecules such as the nanobioconjugates [75]. Once the nanobioconjugates reached the cytoplasm, a rapid endosomal escape took place, which continued in the case of the SH-SY5Y cells. Several studies report that the cationic properties of Buforin II induce a high osmotic pressure within endosomes, leading to an endosomal breach mediated by a proton sponge effect [76,77,78,79]. The present cationic polymers could have aided this process as they might be likely involved in facilitating the proton sponge effect, as reported elsewhere [80]. Our colocalization studies indicate that between 20–40% of nanobioconjugates remained contained in endosomes. At the same time, the rest of them appear reasonably well distributed within cells, as evidenced by the absence of noticeable clusters. Recent reports pointed out that the level of entrapment in other intracellular compartments and organelles is generally insignificant compared with that of endosomes/lysosomes [81,82]. A simple inspection of the obtained images shows that in the case of Vero cells, a fraction of the vehicles was able to reach the nuclear region. This is in agreement with our previous contribution, where we observed the nuclear penetration of the magnetite-PEA-BUFII conjugates [35,43]. However, further experiments will be needed to estimate colocalization within mitochondria and endoplasmic reticulum, which are common regions of interest for the accumulation of exogenous materials [80]. Moreover, because of our interest in gene therapies, we need to explore in detail the ability of our nanobioconjugates to penetrate the nuclear membrane [83].

## 4. Conclusions

The study presented here provides a route for the development of gene delivery systems based on core/shell magnetic nanoparticles, pH-responsive polymers, and translocating peptides. Also, it highlights a robust methodology for the synthesis, functionalization, and loading of plasmid DNA on vehicles equipped with moieties for potent cell penetration. The base core/shell system was successfully synthesized by the coverage of a magnetite core with silver. The obtained magnetite/silver nanoparticles showed an average hydrodynamic diameter of 342 nm. Besides, the superficial charge appears sufficient to assure colloidal stability and prevent DNA degradation. Observation via SEM and TEM confirmed morphology, size, and crystalline structure of the nanoparticles.

The magnetite/silver nanoparticles were further modified with the pH-responsive polymer pDMAEMA, a polyether amine (PEA) surface extensor, and the translocating peptide Buforin II (BUFII). The synthesized magnetite/silver-pDMAEMA-PEA-BUFII nanobioconjugates were characterized with the aid of FTIR and TGA analyses, which corroborated proper conjugation and a peptide conjugation efficiency of about 5%. The ability of the system to form complexes with plasmid DNA was evaluated for the magnetite/silver-pDMAEMA nanoconjugates by loading experiments at pH 8, which showed efficiencies of about 16%. This was followed by the delivery of the cargo material at pH 6.5, which led to efficiencies close to 8%. We determined the saturation limit of the magnetite/silver-pDMAEMA to be 35 ng of DNA per μg of nanobioconjugate for an initial load of 3000 ng (3 μg) of DNA. The DNA-loaded nanoconjugates showed a size change, as evidenced by SEM imaging.

Finally, low cytotoxicity, and hemolytic and platelet aggregation tendencies confirmed the high biocompatibility of the magnetite/silver-pDMAEMA-PEA-BUFII nanobioconjugates and magnetoliposomes (i.e., lecithin liposomes filled with the nanobioconjugates). This is critical to enable the intravenous administration in a subsequent in vivo evaluation stage. Moreover, cell internalization appeared to occur quite rapidly, as evidenced by exceedingly high cytoplasm coverage values in about 30 min, which is considerably faster than most reported cell-penetrating vehicles. Pearson correlation coefficients of colocalization in the range of 20–40% for Vero and neuroblastoma cells corroborated the marked tendency of the synthesized vehicles to escape endosomes. We hypothesized that penetration is proceeded by either clathrin- or caveolin-mediated endocytosis and less probably by direct translocation, while endosome escape is likely to occur by a proton sponge mechanism aided by both BUFII and the conjugated polymers. The results put forward here confirmed that the novel cell-penetrating vehicles have the potential to efficiently deliver nucleotide cargoes, which could be further exploited to overcome some issues associated with gene therapies and particularly those based on CRISPR/Cas9 gene-editing technologies.
